# Agreement between the spatiotemporal gait parameters from two different wearable devices and high-speed video analysis

**DOI:** 10.1371/journal.pone.0222872

**Published:** 2019-09-24

**Authors:** Felipe García-Pinillos, Pedro Á. Latorre-Román, Víctor M. Soto-Hermoso, Juan A. Párraga-Montilla, Antonio Pantoja-Vallejo, Rodrigo Ramírez-Campillo, Luis E. Roche-Seruendo

**Affiliations:** 1 Department of Physical Education, Sports and Recreation, Universidad de La Frontera, Temuco, Chile; 2 Department of Corporal Expression, University of Jaen, Jaen, Spain; 3 Sport and Health University Research Center (iMUDS), University of Granada, Granada, Spain; 4 Department of Pedagogy, University of Jaen, Jaen, Spain; 5 Laboratory of Human Performance, Quality of Life and Wellness Research Group, Department of Physical Activity Sciences, Universidad de Los Lagos, Osorno, Chile; 6 Universidad San Jorge, Villanueva de Gallego, Zaragoza, Spain; University of Brasilia, BRAZIL

## Abstract

This study aimed to evaluate the concurrent validity of two different inertial measurement units for measuring spatiotemporal parameters during running on a treadmill, by comparing data with a high-speed video analysis (VA) at 1,000 Hz. Forty-nine endurance runners performed a running protocol on a treadmill at comfortable velocity (i.e., 3.25 ± 0.36 m.s^-1^). Those wearable devices (i.e., Stryd™ and RunScribe™ systems) were compared to a high-speed VA, as a reference system for measuring spatiotemporal parameters (i.e. contact time [CT], flight time [FT], step frequency [SF] and step length [SL]) during running at comfortable velocity. The pairwise comparison revealed that the Stryd™ system underestimated CT (5.2%, p < 0.001) and overestimated FT (15.1%, p < 0.001) compared to the VA; whereas the RunScribe™ system underestimated CT (2.3%, p = 0.009). No significant differences were observed in SF and SL between the wearable devices and VA. The intra class correlation coefficient (ICC) revealed an almost perfect association between both systems and high-speed VA (ICC > 0.81). The Bland-Altman plots revealed heteroscedasticity of error (*r*^2^ = 0.166) for the CT from the Stryd™ system, whereas no heteroscedasticity of error (*r*^2^ < 0.1) was revealed in the rest of parameters. In conclusion, the results obtained suggest that both foot pods are valid tools for measuring spatiotemporal parameters during running on a treadmill at comfortable velocity. If the limits of agreement of both systems are considered in respect to high-speed VA, the RunScribe™ seems to be a more accurate system for measuring temporal parameters and SL than the Stryd™ system.

## Introduction

The interest in running gait analysis is well justified since an important body of literature has demonstrated its key role in both maximizing athletic performance [1–5] and minimizing the risk of injury [6,7]. In that context, the development of new technologies has allowed researchers to migrate from artificial laboratory settings with expensive systems to more natural environments with low-cost, portable gait analysis equipment [8].

Nowadays, there is a high variety of available technologies for gait analysis (e.g., accelerometers, gyroscopes, force plates, pressure plates, photoelectric cells). Among these, inertial measurement units (IMUs) have gained special popularity among coaches and athletes as low-cost and portable systems. Some wearable devices (e.g., RunScribe™, Stryd™ or Myotest™) provide feedback, even in real-time, on spatiotemporal gait characteristics during running.

Previous studies [9–12] have focused on determining the validity of some wearable devices (i.e. Stryd™ and Myotest™) for measuring stride characteristics during running. García-Pinillos et al. [9] examined the validity of the Stryd™ system compared to OptoGait™, whereas others [10,12] assessed the validity of the Myotest™ system against a photocell-based system [10] and a foot-mounted accelerometer at 1,000 Hz [12]. However, no previous studies have considered the validation of the RunScribe™ system for measuring spatiotemporal parameters, or the comparison of this foot pod with a very similar one (i.e., Stryd™ system), which represents a very popular alternative among practitioners.

While the Stryd™ system (Stryd Powermeter, Stryd Inc. Boulder CO, USA) is a foot pod that weights 9.1 grams and it is based on a 6-axis IMU (3-axis gyroscope, 3-axis accelerometer), the RunScribe™ system (Scribe Lab. Inc. San Francisco CA, USA) weights 15 g and it is based on a 9-axis (3-axis gyroscope, 3-axis accelerometer, 3-axis magnetometer), with a sampling rate of 500 Hz. Both systems provide different metrics to quantify intensity and running biomechanics.

Since the validity and reliability of a gait analysis system are essential to determine whether results are due to changes in gait pattern or are simply systematic measurement errors, the limitation is not how to collect the data but how valid these parameters are. Therefore, the aim of this study is to evaluate the concurrent validity of two different foot pods (i.e., Stryd™ and RunScribe™) for measuring spatiotemporal parameters during running on a treadmill at a comfortable velocity, by comparing data with a high-speed video analysis (VA) at 1,000 Hz as a reference system.

## Materials and methods

### Participants

Men (n = 44) and women (n = 5) amateur endurance runners (age: 26±8 years; height: 1.74±0.07 m; body mass: 71±10 kg) participated in this study. All participants met the inclusion criteria: (1) older than 18 years, (2) able to run 10 km in less than 50 minutes, (3) not suffering from any injury in the last 6 months before the data collection. After receiving detailed information on the objectives and procedures of the study, each participant signed an informed consent form in order to participate, which complied with the ethical standards of the World Medical Association’s Declaration of Helsinki (2013). It was made clear that the participants were free to leave the study if they saw fit. The Institutional Review Board approved the study.

### Procedures

Participants were individually tested on one specific day. Prior to all testing, subjects refrained from severe physical activity for at least 48 h and all tests were completed ≥3 h after eating. Tests were performed with the subjects’ usual training shoes checking that corresponds to an A3 running shoes category to measure their typical performance [13].

Participants performed a running protocol on a motorized treadmill (WOODWAY Pro XL, Woodway, Inc., Waukesha, WI, USA). Treadmill speed was calibrated following Padulo’s proposal [13]. The difference between treadmill speed calibrated and showed in the deck was 0,04%. The initial speed was set at 2.22 m.s^−1^, and speed increased by 0.28 m.s^−1^ every minute until participants felt comfortable. Then, running velocity was fixed (i.e. self-selected comfortable running velocity: 3.25 ± 0.36 m.s^-1^). Since previous studies [14,15] on human locomotion have shown that accommodation to running on a treadmill occurs in ~6–8 min, an 8 min accommodation interval was performed at the aforementioned self-selected velocity. Immediately after the accommodation interval, the recording period (i.e., 3 min) started, performed at the self-selected comfortable running velocity. The slope was maintained at 0% over the entire protocol.

### Materials and testing

For descriptive purposes, height (m) and body mass (kg) were measured using a precision stadiometer and scale (SECA 222 and 634, respectively, SECA, Corp., Hamburg, Germany).

Spatiotemporal parameters during running were considered [13]: contact time (CT, in seconds): time from the first frame when the foot contacts the ground to last shoe contact before the toes lift off the ground; flight time (FT, in seconds): time from toe-off to initial ground contact of consecutive footfalls (e.g., right-left); step length (SL, in meters): length or distance from one foot strike to the next foot strike of the opposite foot; step frequency (SF, in steps/min): number of ground contact events per minute. Three different systems were used simultaneously to measure those parameters: two different wearable devices (i.e., Stryd™ and RunScribe™ systems) and a high-speed VA (1,000 Hz), this last one as the reference method. In order to assess CT and FT in speeds higher than 5.56 m·s^-1^ at least a 250 fps sampling rate has been proposed [13]. Of note, for the analysis of CT, FT, SL and SF parameters the right leg was always the analyzed leg in order to control potential influencing factors (i.e., asymmetry [16]). Further information about the systems:

- Stryd™ (Stryd Powermeter, Stryd Inc. Boulder CO, USA): It is a carbon fibre-reinforced foot pod (attached to the lace shoe of the right leg) that weights 9.1 grams. Despite being a relatively new tool, some studies have already examined its validity and reliability for measuring spatiotemporal parameters [9] and the association of power output with running economy [17]. Based on a 6-axis IMU (3-axis gyroscope, 3-axis accelerometer), this device provides twelve metrics to quantify performance: pace, distance, elevation, running power, form power, SF, ground CT, vertical oscillation and leg stiffness. Based on a previous study [9], from CT, SF and running velocity, the authors calculated FT and SL as follows:
  FT(s)=steptime(s)−CT(s) (1)
  where step time (ST) is the time from the beginning of the step cycle (foot strike) to the end (previous frame to the foot strike of the same side),
  $$ST\;\left(s\right)=60/SF\;\left(steps.min^{‐1}\right)$$
  $$SL\;\left(m\right)=running\;velocity\;\left(m.min^{‐1}\right)/SF\;\left(steps.min^{‐1}\right)$$ (2)
- RunScribe™ system (Scribe Lab. Inc. San Francisco CA, USA): It is an IMU based on 9-axis (3-axis gyroscope, 3-axis accelerometer, 3-axis magnetometer), with a sampling rate of 500 Hz (accuracy of 0.002 s). It was also attached to the lace shoe of the right leg. Results from RunScribe™ were taken from their website (https://dashboard.runscribe.com/runs) into the .csv file. Then, data were imported from Excel® and analyzed. Means were calculated for spatiotemporal parameters from the 3 minutes recorded, and a 30 s window, from 1:30 to 2:00 min, was analyzed.
- Video analysis: Two-dimensional video data were simultaneously collected at 1,000 Hz using a high-speed camera (Imaging Source DFK 33UX174, The Imaging Source Europe GmbH; Germany). Range of interest (ROI) was adjusted to achieve 1,000 fps (784x144 resolution). The camera was placed perpendicular to the treadmill from a posterior view at 2 m from the center of the treadmill and at a height of 0.80 m. The 30 s videos were recording between 1:30–2:00 min of the recording period of each participant. Then, videos were analysed using the open license software Kinovea (version 0.8.27), and spatiotemporal parameters were determined. The CT and FT were calculated by identifying both the initial contact and the take-off frames and counting frames in-between; whereas SL and SF were calculated as follows:
  ST(s)=FT(s)+CT(s), (3)
  $$SF\;\left(steps.s^{‐1}\right)=1/ST\;\left(s\right)$$ (4)
  $$SF\;\left(steps.min^{‐1}\right)=60\;x\;SF\;\left(steps.s^{‐1}\right)$$ (5)
  $$SL\;\left(m\right)=running\;velocity\;\left(m.min^{‐1}\right)/SF\;\left(steps.min^{‐1}\right)$$ (6)

### Statistical analysis

Descriptive statistics are represented as mean standard deviation (SD). Tests of normal distribution and homogeneity, determined by the Shapiro-Wilk and Levene’s test, respectively, were conducted on all data before analysis. To determine concurrent validity, a Pearson correlation analysis was performed between spatiotemporal parameters from wearable devices and VA. The following criteria were adopted to interpret the magnitude of correlations between measurement variables: <0.1 (trivial), 0.1–0.3 (small), 0.3–0.5 (moderate), 0.5–0.7 (large), 0.7–0.9 (very large) and 0.9–1.0 (almost perfect) [18]. Intra class correlation coefficients (ICC) were also calculated between systems (Stryd™ vs. VA, and RunScribe™ vs. VA) for spatiotemporal parameters during running. Based on the characteristics of this experimental design and following the guidelines reported by Koo and Li [19], the authors decided to conduct a “two-way random-effects” model (ICC [2,k]), “mean of measurements” type, and “absolute” definition for the ICC measurement. The interpretation of the ICC was based on the benchmarks reported by a previous study [20]: ICC < 0 (poor), 0–0.20 (slight), 0.21–0.40 (fair), 0.41–0.60 (moderate), 0.61–0.80 (substantial), and > 0.81 (almost perfect). Pairwise comparisons of means (t-test) were also conducted between data from the aforementioned systems (Stryd™ vs. VA, and RunScribe™ vs. VA). The magnitude of the differences between values was also interpreted using the Cohen’s d effect size (*ES*) (between-group differences) [21]. Effect sizes are reported as: trivial (<0.19), small (0.2–0.49), medium (0.5–0.79), and large (≥0.8) [21]. Finally, Bland-Altman plots (i.e., limits of agreement method, mean difference ± 1.96 SD) [22] were constructed to examine the presence of systematic and proportional bias between VA at 1,000 Hz and estimated values (i.e. two wearable devices) of spatiotemporal parameters during running. Heteroscedasticity of error was defined as an *r*^2^>0.1 [23]. The level of significance used was p<0.05. Data analysis was performed using SPSS (version 23, SPSS Inc., Chicago, Ill).

## Results

The pairwise comparison between wearable devices and VA revealed some significant differences (Table 1). The Stryd™ system underestimated CT (5.2%, p < 0.001) and overestimated FT (15.1%, p < 0.001) with medium ES (~0.5–0.6) compared to the VA; whereas the RunScribe™ system underestimated CT (2.3%, p = 0.009) with small ES (< 0.21).

**Table 1:** Means comparison of spatiotemporal parameters obtained from Stryd™ and RunScribe™ systems compared to a reference system.

Variable	Stryd	RunScribe	VA	Stryd vs. VA Δ (%)	RunScribe vs. VA Δ (%)	Stryd vs. VA *p-value (ES)*	RunScribe vs. VA *p-value (ES)*
CT (s)	0.253 (0.022)	0.261 (0.028)	0.267 (0.028)	0.014 (5.2%)	0.006 (2.3%)	<0.001 (0.56)	0.009 (0.21)
FT (s)	0.107 (0.023)	0.096 (0.026)	0.093 (0.025)	-0.014 (15.1%)	-0.003 (3.2%)	<0.001 (0.58)	0.182 (0.12)
SF (spm)	166.72 (7.26)	168.13 (7.42)	166.81 (7.69)	0.09 (0.1%)	-1.32 (0.8%)	0.823 (0.01)	0.071 (0.18)
SL (cm)	118.05 (13.47)	116.34 (12.12)	116.89 (12.50)	-1.16 (1.0%)	0.55 (0.5%)	0.077 (0.09)	0.378 (0.05)

Δ: between systems differences (VA–foot pod); VA: High-speed video analysis at 1,000 Hz; CT: contact time; FT: flight time; SF: step frequency; SL: step length; ES: Cohen´s d effect size.

The Pearson correlation analysis reported significant relationships between systems in every variable (Table 2). In both comparisons (i.e., Stryd™ and RunScribe™ vs. VA), very large correlations (r > 0.75) were obtained in CT and FT, with almost perfect correlations found in SF and SL (r > 0.93). Likewise, both comparisons (i.e., Stryd™ and RunScribe™ vs. VA) revealed almost perfect associations in terms of ICC (> 0.81) (Table 2).

**Table 2:** Pearson correlation analysis (r) and intraclass correlation coefficients (ICC) between spatiotemporal parameters during running obtained from Stryd™ and RunScribe™ systems compared to a reference system (high-speed VA).

Variables	Stryd vs. VA			RunScribe vs. VA		
	Coefficient (*r*)	*p-value*	*ICC* *(95% CI)*	Coefficient (*r*)	*p-value*	*ICC* *(95% CI)*
Contact time	0.820	< 0.001	0.813 (0.292–0.927)	0.831	< 0.001	0.896 (0.799–0.944)
Flight time	0.809	< 0.001	0.807 (0.179–0.929)	0.754	< 0.001	0.857 (0.747–0.920)
Step frequency	0.932	< 0.001	0.965 (0.937–0.980)	0.945	< 0.001	0.964 (0.915–0.983)
Step length	0.938	< 0.001	0.975 (0.955–0.986)	0.957	< 0.001	0.968 (0.943–0.982)

VA: High-speed video analysis at 1,000 Hz; ICC: intra class correlation coefficients; CI: confidence interval

**Fig 1:**
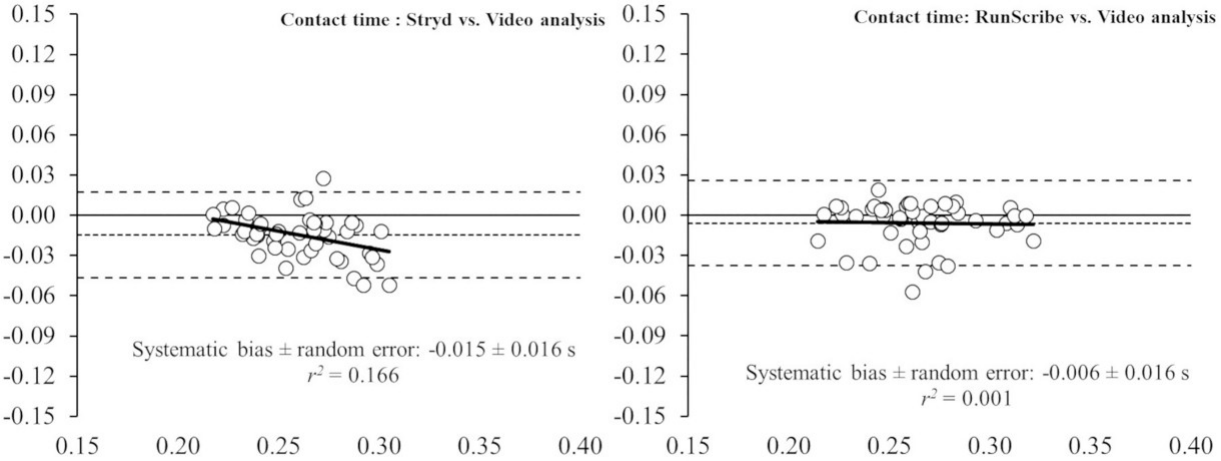
Bland-Altman plots for the measurement of contact time (CT) during running from Stryd™ and RunScribe™ systems compared to high-speed video analysis. The plot includes the mean difference (dotted line) and 95% limits of agreement (dashed lined), along with the regression line (solid line).

**Fig 2:**
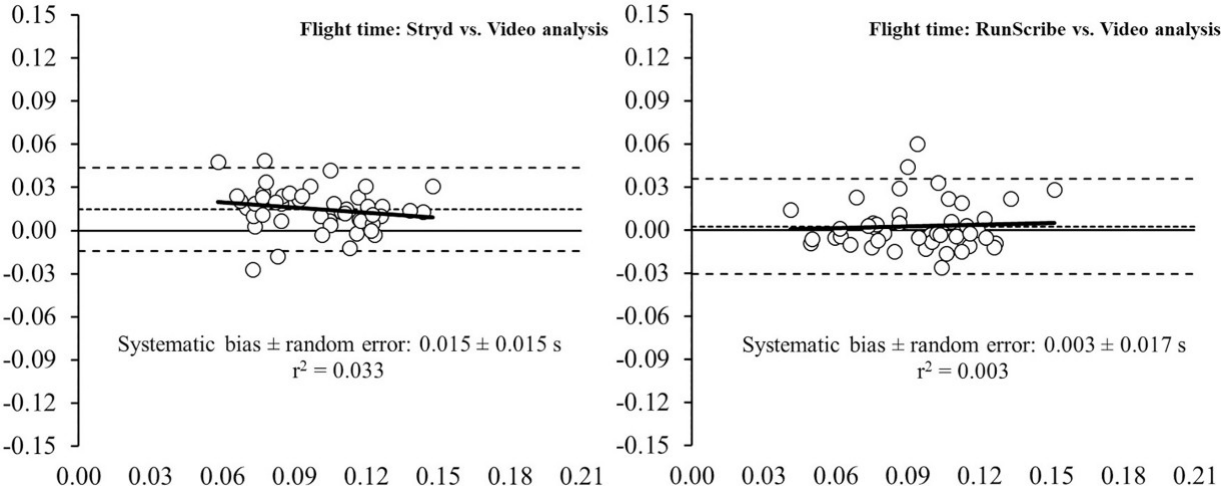
Bland-Altman plots for the measurement of flight time (FT) during running from Stryd™ and RunScribe™ systems compared to high-speed video analysis. The plot includes the mean difference (dotted line) and 95% limits of agreement (dashed lined), along with the regression line (solid line).

**Fig 3:**
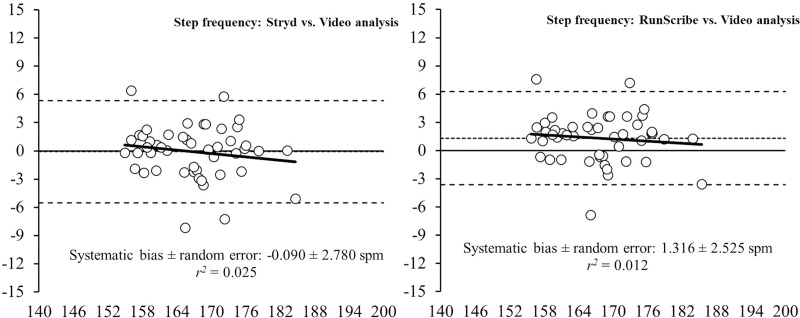
Bland-Altman plots for the measurement of step frequency (SF) during running from Stryd™ and RunScribe™ systems compared to high-speed video analysis. The plot includes the mean difference (dotted line) and 95% limits of agreement (dashed lined), along with the regression line (solid line).

**Fig 4:**
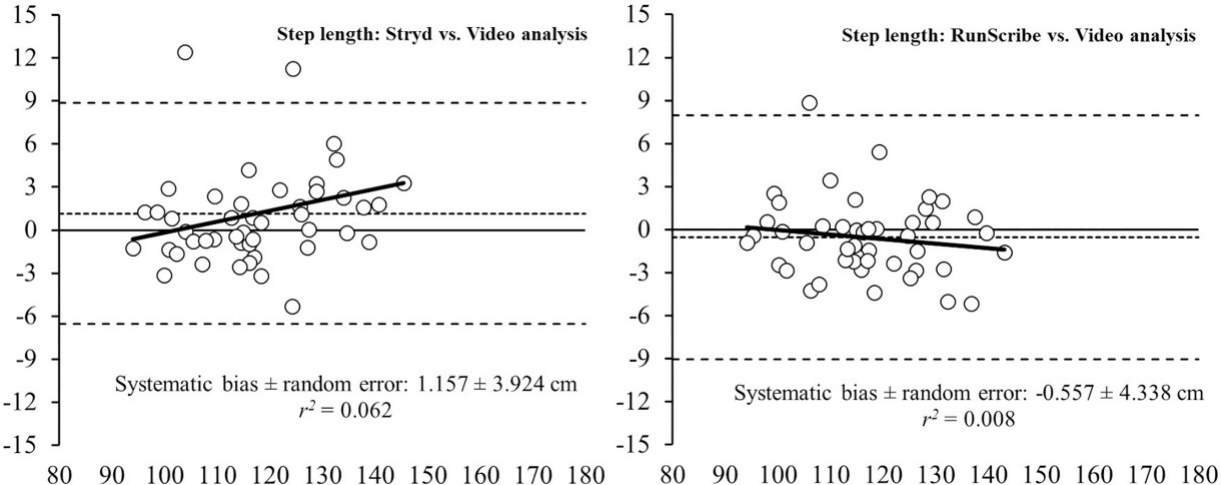
Bland-Altman plots for the measurement of step length (SL) during running from Stryd™ and RunScribe™ systems compared to high-speed video analysis. The plot includes the mean difference (dotted line) and 95% limits of agreement (dashed lined), along with the regression line (solid line).

Bland-Altman plots (Figs 1–4) show the between-system differences compared to high-speed VA (i.e., Stryd™ and RunScribe™ vs. VA) and the degree of agreement between the systems (95% limits of agreement). These plots revealed heteroscedasticity of error (*r*^2^ = 0.166) for the CT from the Stryd™ system, whereas no heteroscedasticity of error (*r*^2^ < 0.1) was revealed in the rest of parameters. Compared to data from VA, the systematic bias was higher in data from the Stryd™ system for the CT (Fig 1), FT (Fig 2) and SL (Fig 4), (i.e., wider limits of agreement than RunScribe™ system), while the systematic bias was higher in SF (Fig 3) from the RunScribe™ system (i.e., wider limits of agreement than Stryd™ system).

## Discussion

This study aimed to determine the level of agreement between two different foot pods (i.e., Stryd™ and RunScribe™ systems) and a high-speed VA at 1,000 Hz for measuring spatiotemporal parameters during running. Although the ICCs revealed an almost perfect association between both systems and high-speed VA (ICC > 0.81), the pairwise comparisons showed some differences with the Stryd™ system underestimating CT (5.2%) and overestimating FT (15.1%), while the RunScribe™ system underestimated CT (2.3%). The Bland-Altman plots build up that analysis showing that the systematic bias was higher in the Stryd™ system for the CT, FT and SL, while the systematic bias was higher in SF from the RunScribe™ system (always compared to high-speed VA data).

Having the chance to measure athletes or clients in a natural environment and using less expensive and more time-efficient equipment is a huge step forward for coaches and clinicians [8]. Nevertheless, this advantage would be worthless if the data were not valid. Both RunScribe™ and Stryd™ systems are running power meters, but also provide spatiotemporal variables that are used by coaches and clinicians (information easily accessible to users) as a feedback, necessitating confirmation of the validity of these data.

In that context, and given the increased popularity of these systems, previous studies have determined the validity of some wearable devices for measuring stride characteristics during running [9–12]. As earlier mentioned, García-Pinillos et al. [9] examined the agreement between spatiotemporal parameters from Stryd™ system vs. OptoGait™ system (the later as the reference system) during running at different velocities, and the authors concluded that Strydx system provides accurate SL and SF measures but underestimates CT (0.5–8%) and overestimates FT (3–67%) compared to the reference system. Despite methodological differences (i.e., OptoGait™ system vs. high-speed VA as the methods of reference), the results reported by the current study are in line with those reported by García-Pinillos et al. [9] at similar running velocities (~3.3 m.s^-1^) with the Stryd™ system underestimating CT (5.2%), overestimating FT (15.1%) and providing accurate SL and SF (differences lower than 1%) compared to high-speed VA. However, differences in the magnitude of current results were noticed when compared to those from García-Pinillos et al. [9], which might be related to the characteristics of the OptoGait™ system. It is based on the communication between two photoelectric cell bars configured with the LEDs 3mm from the ground, which results in the LEDs being interrupted a few milliseconds before contact with the ground and a few milliseconds after foot off (i.e., longer stance phase and shorter swing phase when compared to other gait analysis systems [24–26].

Regarding the RunScribe™, no previous studies have analysed its validity. However, previous studies examined the validity of wearable devices for measuring stride characteristics during running [10–12]. Gindre et al. [10] assessed the validity of the Myotest™ system against the OptoJump™ system (i.e., reference method), observing shorter CT (34%) and longer FT (64%) with the Myotest™ system. Gouttebarge et al. [12] compared the Myotest™ system against a foot-mounted accelerometer (at 1,000 Hz) and the authors reported an accurate estimation of SF (< 1% difference) but great between-system differences in CT (-175%) in endurance runners. Since the RunScribe™ system reported smaller differences (i.e., 2.3% in CT, -3.2% in FT and < 1% in SF and SL) compared to the reference system used in the current work, the results suggest that this system is a more accurate device for measuring spatiotemporal parameters.

Since methodological differences can be observed among the aforementioned studies, some points must be considered to properly interpret these comparisons. Whereas the current work used a high-speed VA system (1,000 Hz) as a gold-standard, previous works used photoelectric cell-based systems (i.e., OptoGait™ system [9] and OptoJump™ system [10], force platforms [11] or accelerometry [12], which might have an influence on the differences reported. Additionally, the placement of the wearables is not consistent between studies and it seems to be system-dependent. Whereas the Stryd™ system was tested when attached onto the lace shoe [9], the Myotest™ system has been tested in different placements (i.e. waist [10] or lace shoe [11,12]). The current study focused on determining the accuracy of two different systems attached to the lace shoe for estimating spatiotemporal parameters during running.

In the current study, two different foot pods were placed in the same lace shoe of participants to collect data simultaneously during running, and those systems were compared to the same reference system (i.e., high-speed VA at 1,000 Hz). Such protocol allow an indirect comparison of the accuracy for measuring spatiotemporal parameters of both foot pods compared to the same reference system. The results from pairwise comparisons (Stryd™ and RunScribe™ vs. VA) revealed greater differences for the Stryd™ system (i.e., -5.2% in CT and +15.1% in FT) than the RunScribe™ system (i.e., -2.3% in CT and +3.2% in FT). Additionally, the Bland-Altman plots reinforced that finding with higher systematic bias in data from the Stryd™ system for the CT, FT and SL (i.e., wider limits of agreement than RunScribe™ system). Therefore, the results suggest that the RunScribe™ system is more accurate than the Stryd™ system for estimating spatiotemporal parameters during running, as compared to a high-speed VA.

Finally, some considerations must be taken into account. First, these results could be restricted to amateur endurance runners [5,27] and to a running protocol performed on a treadmill at a comfortable velocity [28]. Second, the footwear was not standardized, but all runners wore their own footwear to increase the ecological validity of the study. Third, the criterion measure used. Even though the high-speed VA has been shown to be a reliable and valid method to measure running kinematics [29–32], the 3-D motion capture system is widely considered as a `gold-standard´ for this purpose. Fourth, reliability data were not reported in the current study so, findings cannot be generalised to runs performed several days apart. Notwithstanding those points, the current study provides some insights into the validity of spatiotemporal parameters assesses from two new systems (i.e. Stryd™ and RunScribe™), by using a high-speed VA at 1,000 Hz as the gold standard, with a high frequency and high resolution, installed at surface level, which allowed a great accuracy for determining spatiotemporal parameters.

## Conclusions

In conclusion, both foot pods (i.e., Stryd™ and RunScribe™) are valid tools for measuring spatiotemporal parameters during running on a treadmill at comfortable velocity. If the limits of agreement of both systems are considered in respect to high-speed VA, the RunScribe™ seems to be a more accurate system for measuring temporal parameters and step length than the Stryd™ system.

From a practical standpoint, the differences reported in both devices as compared to high-speed VA warn coaches, clinicians and scientists about the bias of comparing data from different devices (i.e., Stryd™ system and RunScribe™ system).

## Supporting information

S1 Database (XLSX)
